# Regional outcomes of severe acute respiratory syndrome coronavirus 2 infection in hospitalised patients with haematological malignancy

**DOI:** 10.1111/ejh.13469

**Published:** 2020-07-13

**Authors:** Stephen Booth, John Willan, Henna Wong, Dalia Khan, Rachel Farnell, Alicia Hunter, Toby Eyre, Harley Katz, Moez Dungarwalla, Lucia Chen, Joe Browning, Paolo Polzella, Nicola Gray, Pratap Neelakantan, Elissa K. Dhillon, David Dutton, Alex Sternberg, Steven Prideaux, Graham P. Collins, Andy Peniket

**Affiliations:** ^1^ Department of Haematology Oxford University Hospitals NHS Foundation Trust Oxford UK; ^2^ Department of Haematology Frimley Health NHS Foundation Trust Frimley UK; ^3^ Radcliffe Department of Medicine School of Clinical Medicine University of Oxford Oxford UK; ^4^ Astrophysics Department University of Oxford Oxford UK; ^5^ Department of Haematology Milton Keynes University Hospital NHS Foundation Trust Milton Keynes UK; ^6^ Department of Haematology Buckinghamshire Healthcare NHS Trust High Wycombe UK; ^7^ Department of Haematology Royal Berkshire Hospital NHS Foundation Trust Reading UK; ^8^ Department of Haematology Great Western Hospital NHS Foundation Trust Swindon UK

**Keywords:** haematological malignancy, SARS‐CoV‐2

## Abstract

**Objectives:**

We sought to characterise the outcomes of patients with haematological malignancy and severe acute respiratory syndrome coronavirus 2 (SARS‐CoV‐2) infection in hospital in our regional network of 7 hospitals.

**Methods:**

Consecutive hospitalised patients with haematological malignancy and SARS‐CoV‐2 infection were identified from 01/03/2020 to 06/05/2020. Outcomes were categorised as death, resolved or ongoing. The primary outcome was preliminary case fatality rate (pCFR), defined as the number of cases resulting in death as a proportion of all diagnosed cases. Analysis was primarily descriptive.

**Results:**

66 Patients were included, overall pCFR was 51.5%. Patients ≥ 70 years accounted for the majority of hospitalised cases (42, 63%) and fatalities (25, 74%). Mortality was similar between females (52%) and males (51%). Immunosuppressive or cytotoxic treatment within 3 months of the diagnosis of SARS‐CoV‐2 infection was associated with a significantly higher pCFR of 70%, compared with 28% in those not on active treatment (*P* = .0013, 2 proportions *z* test).

**Conclusions:**

Mortality rates in patients with haematological malignancy and SARS‐CoV‐2 infection in hospital are high supporting measures to minimise the risk of infection in this population.

**Key points**

1. What is the new aspect of your work?

Our data provide one of the largest series of outcome data for hospitalised patients with haematological malignancy and severe acute respiratory syndrome coronavirus 2 (SARS‐CoV‐2) infection published to date.

2. What is the central finding of your work?

Mortality is high at 51.5% overall and significantly higher in those who received immunosuppressive or cytotoxic treatment in the last three months (70%) than in those who did not (28%).

3. What is (or could be) the specific clinical relevance of your work?

The data support the use of strict measures to protect the population with haematological malignancy from SARS‐Cov‐2 infection.

## 1 INTRODUCTION

The pandemic spread of COVID‐19 (coronavirus disease 2019) caused by severe acute respiratory syndrome coronavirus 2 (SARS‐CoV‐2) has so far caused over 400 000 confirmed deaths worldwide. Preliminary pre‐print data from 16 749 hospitalised patients in the United Kingdom (UK) suggest a mortality of at least 33% in unselected hospitalised patients.^1^ Risk factors associated with more severe outcomes include increasing age, pre‐existing lung disease, diabetes, hypertension and cancer.^2^ The risk of SARS‐CoV‐2 to patients with cancer, and particularly haematological malignancy, is not yet fully clarified.

Early data suggested that patients with cancer are at increased risk of death if contracting this virus.^3^, ^4^, ^5^, ^6^ However, numbers of patients with malignancy were low with marked heterogeneity of diagnoses, and little information was presented on treatment history. With regard to haematological malignancies, a cohort study from Wuhan, China, identified 11 patients with haematological malignancy and COVID‐19, of whom 8 (72%) did not survive.^4^ A further study of 25 patients, of whom 24 had a malignant haematological diagnosis, reported a one‐month mortality rate of COVID‐19 infection of 40%.^7^

Two recent multicentre retrospective cohort studies of patients with COVID‐19 compared patients with cancer with age‐matched controls without cancer. Both of these studies have suggested that patients with haematological cancers may be at greatest risk of severe complications of COVID‐19 when compared with other malignancies. The first study reported a higher odds ratio of death of 2.3 in 105 patients with cancer compared to age‐matched controls.^8^ The 9 patients with haematological malignancy had the worst outcomes, with significantly increased risk of intensive care admission, need for ventilation and death. The second cohort study reported on 218 COVID‐positive patients with a malignant diagnosis, reporting an increased case fatality rate of 2‐3 compared to age‐matched controls.^9^ The 54 patients with haematological malignancy had a case fatality rate of 37%, worse than that of solid organ cancers.

Patients with haematological cancers are likely to be at high risk of infectious complications of viral respiratory infections from both immune dysregulation as an intrinsic part of the malignancy, as well as of the immunosuppressive and cytotoxic treatment.^10^ In time, it will be important to identify from large data sets the diagnoses and treatments which convey the greatest risk to this patient group, and large prospective studies are underway to assemble this information.

We present outcome data for all hospitalised COVID‐19 positive patients with a diagnosis of haematological malignancy from our region, which includes seven hospitals serving a population of 2.8 million patients,^11^ as a first attempt to understand in more detail the outcome of these patients.

## 2 METHODS

Consecutive cases were identified prospectively by clinical teams across our regional cancer network from 01/03/2020 to 06/05/2020 and reported to a central database. Patients were required to be hospitalised and positive for SARS‐CoV‐2 RNA by reverse transcriptase quantitative polymerase chain reaction (qPCR) of nose and throat swab, or with clinical and radiological features consistent with COVID‐19, where the clinical team judged COVID‐19 was the most likely diagnosis. All patients had a current haematological malignancy under ongoing treatment or in clinical follow‐up. COVID‐19 was treated according to local practice with many patients entering clinical trials.

Patients who were not admitted to hospital were not included in the analysis because of variation in outpatient testing strategy over time and between hospitals. Patients with asymptomatic non‐malignant conditions, for example monoclonal gammopathy of uncertain significance, were excluded.

Patient baseline characteristics collected included age, gender, haematological diagnosis, method of diagnosis of SARS‐CoV‐2, current haematological treatment and prior lines of treatment.

The primary outcome was preliminary case fatality rate (pCFR), defined as the number of cases resulting in death as proportion of all diagnosed cases.^12^ Outcomes were categorised as either death; resolved (patients who were no longer symptomatic and judged to have recovered from the infection by their clinical team); or ongoing (patients remained in hospital with symptoms attributed to SARS‐CoV‐2 infection).

Analysis was primarily descriptive with the two proportions *Z* test used to compare pCFR in the population of patients who received immunosuppressive or cytotoxic treatment in the 3 months prior to SARS‐CoV‐2 infection and the population who did not receive such treatment.

## 3 RESULTS

A total of 66 hospitalised patients with a haematological malignancy diagnosed with SARS‐CoV‐2 infection were identified by clinical teams. Baseline characteristics were as follows: median age 73 years (interquartile range [IQR] 63‐81 years), gender: 41 (62%) male, 25 (38%) female; 37 (56%) of the patients had received immunosuppressive or cytotoxic treatment within 3 months of diagnosis with SARS‐CoV‐2 infection. 61 (92%) patients were diagnosed by positive SARS‐CoV‐2 reverse transcriptase PCR, 5 (8%) were diagnosed by radiological and clinical features where it was felt the nasopharyngeal swab was giving a false negative result. At the time of data cut‐off, median survival follow‐up was 32.5 days across all patients and 61.5 days for patients who had not died, including 28 patients with resolved infection and 4 patients with ongoing symptoms in hospital.

Haematological diagnoses were as follows: acute myeloid leukaemia (AML) 8 (12%), myelodysplastic syndrome (MDS) or chronic myelomonocytic leukaemia (CMML) 8 (12%), myeloproliferative neoplasms (MPNs) 5 (8%), myeloma 17 (26%), lymphoma 15 (23%), chronic lymphocytic leukaemia (CLL) 11 (17%), T‐cell large granular lymphocytic leukaemia T‐(LGL) 2 (3%).

**FIGURE 1:**
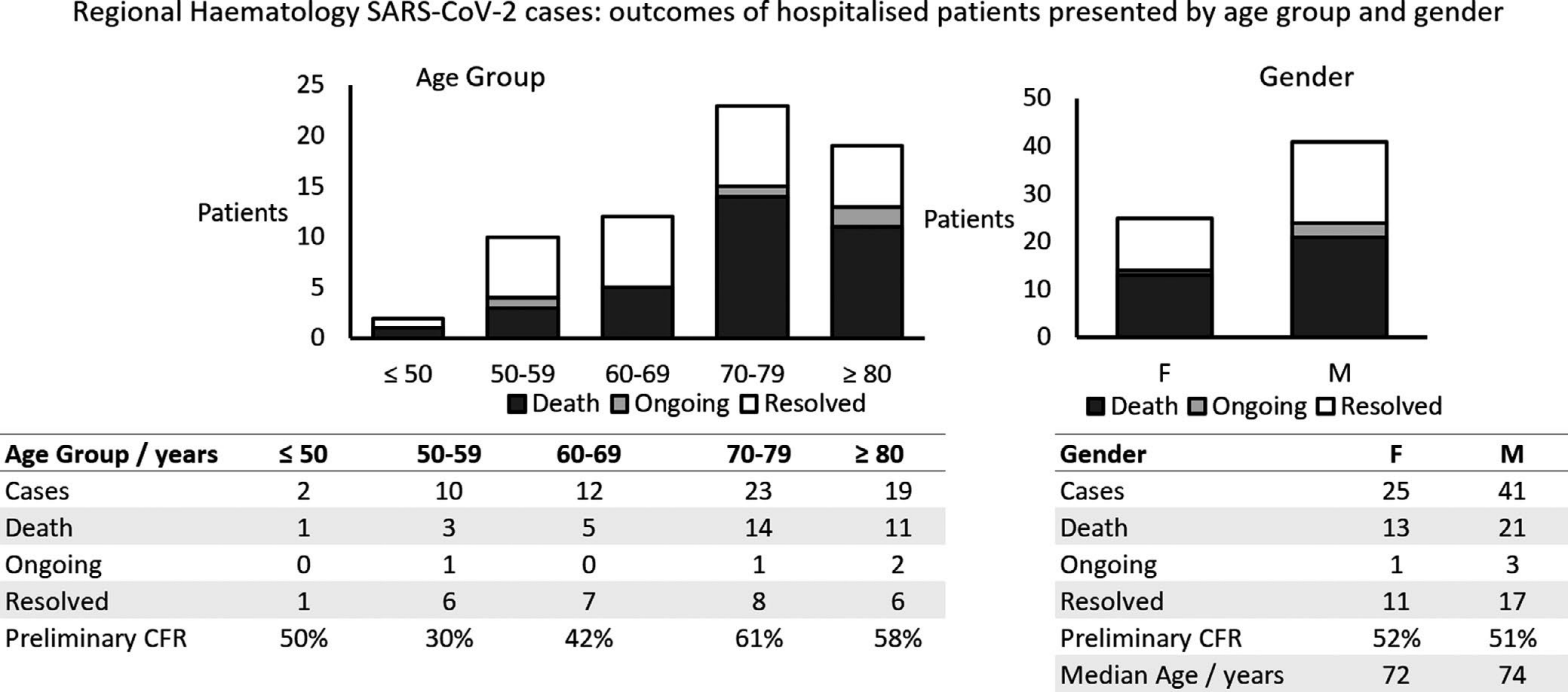
Regional outcomes of hospitalised patients with haematological malignancy and severe acute respiratory syndrome coronavirus 2 infection by age and gender. Outcomes shown categorised as death, ongoing symptomatic infection in hospital and resolved. CFR, case fatality rate defined as deaths as a proportion of all cases; F, female; M, male

**FIGURE 2:**
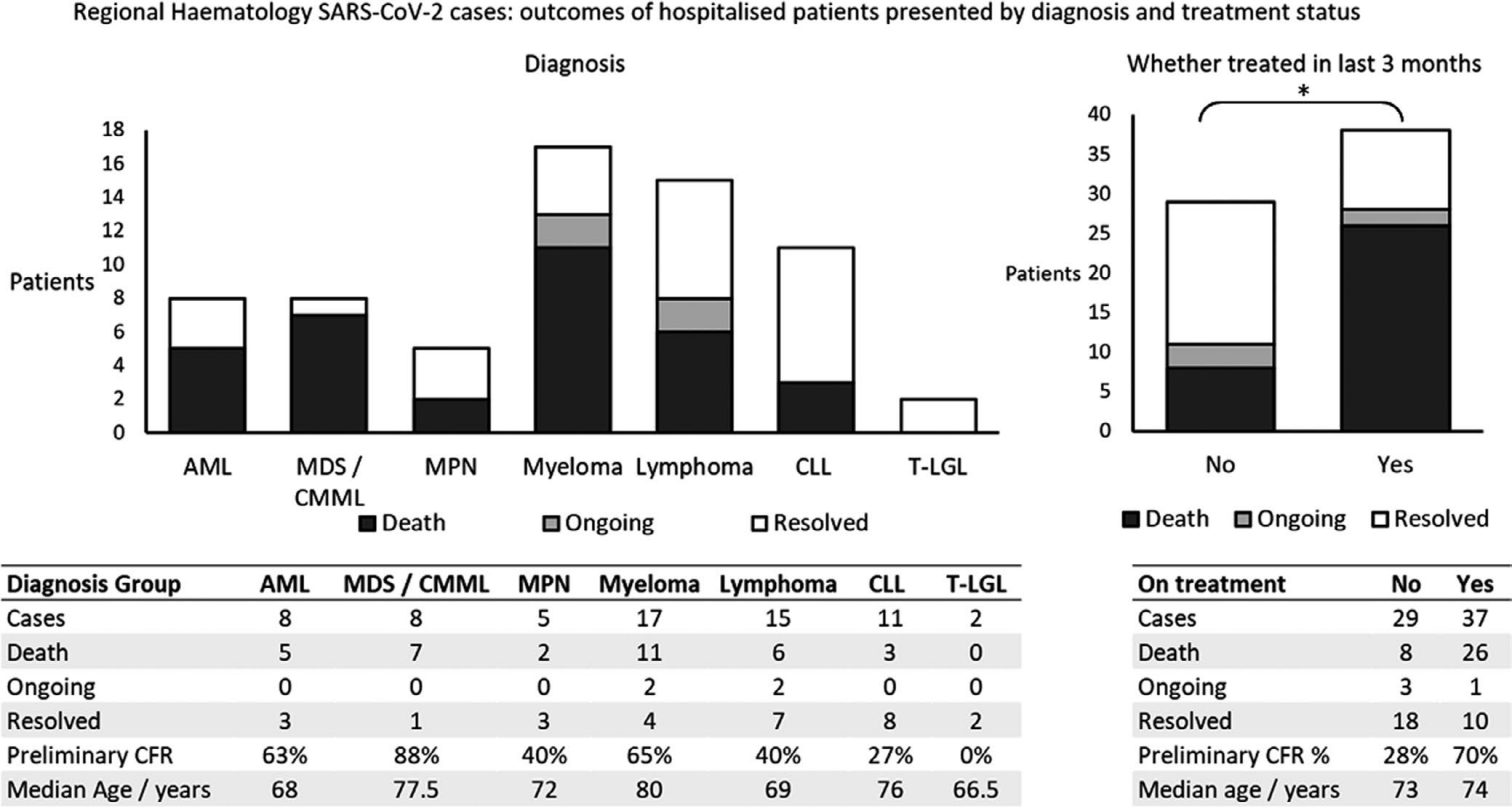
Regional outcomes of hospitalised patients with haematological malignancy and severe acute respiratory syndrome coronavirus 2 infection by haematology diagnosis and whether given systemic immunosuppressive or cytotoxic treatment in the last 3 months. Outcomes shown categorised as death, ongoing symptomatic infection in hospital, and resolved. Preliminary CFR, defined as deaths as a proportion of all cases, was significantly higher in patients receiving treatment *P* = .0013, with difference in pCFR 0.426 ± 0.184 (90% confidence interval, 2 proportions *Z* test). AML, acute myeloid leukaemia; CFR, case fatality rate; CLL, chronic lymphocytic leukaemia; CMML, chronic myelomonocytic leukaemia; MDS, myelodysplastic syndrome; T‐LGL T‐cell large granular lymphocytic leukaemia

In total, there were 34 deaths; therefore, the overall pCFR was 51.5%, with patients over the age of 70 accounting for the majority of cases 42 (64%) and fatalities 25 (74%) (Figure 1). Numbers of deaths in each age group were as follows: <60 years, 4; 60‐69 years, 5; 70‐69 years 14; >80 years, 11. The pCFR was similar between female and male patients (52% and 51%, respectively). Mortality rates were consistently high across diagnostic groups, particularly the myeloid malignancies and myeloma (Figure 2): AML 5 (63%), MDS or CMML 7 (88%), MPNs 2 (40%), myeloma 11 (65%), lymphoma 6 (40%) and CLL 3 (27%). Detailed data on patient characteristics and treatment received are provided in Table 1.

**TABLE 1:** Characteristics and outcomes of regional hospitalised patients with haematological malignancy and severe acute respiratory syndrome coronavirus 2 infection

Case no.	Diagnosis	Method of SARS‐CoV‐2 diagnosis	Age group/years	Gender	Therapy Y/N	Therapy within 3 mo	Days from last therapy to SARS‐CoV‐2 diagnosis	Prior therapies	Respiratory support	Outcome
1	AML	RT‐PCR	60‐69	M	Y	DA induction	16	N/A	NIL	Resolved
2	AML	RT‐PCR	≥80	F	Y	Hydroxycarbamide	1	N/A	O_2_	Death
3	AML	RT‐PCR	50‐59	M	Y	Liposomal DA	23	N/A	CPAP	Death
4	AML	RT‐PCR	≥80	F	Y	Hydroxycarbamide	1	N/A	O_2_	Death
5	AML	RT‐PCR	70‐79	M	Y	Azacitadine	10	DA	O_2_	Death
6	AML	RT‐PCR	60‐69	F	NIL	NIL	N/A	N/A	NIL	Death
7	AML	RT‐PCR	50‐59	M	NIL	NIL	N/A	N/A	O_2_	Resolved
8	T‐AML	RT‐PCR	60‐69	F	NIL	NIL	N/A	N/A	O_2_	Resolved
9	CMML	RT‐PCR	70‐79	F	Y	Hydroxycarbamide	1	N/A	O_2_	Death
10	MDS	RT‐PCR	70‐79	F	Y	Azacitadine	2	N/A	NIL	Death
11	T‐MDS	CT	70‐79	M	Y	Venetoclax & Azacitadine	14	Azacitadine & Magrolimab, R‐Bendamustine, FCR	NIL	Death
12	T‐MDS	CT	70‐79	M	Y	Azacitadine	28	N/A	O_2_	Death
13	CMML	RT‐PCR	≥80	M	NIL	NIL	N/A	N/A	NIL	Death
14	MDS	RT‐PCR	60‐69	M	NIL	NIL	N/A	Azacitadine	O_2_	Death
15	MDS	RT‐PCR	≥80	M	NIL	NIL	N/A	N/A	O_2_	Death
16	MDS	RT‐PCR	≥80	M	NIL	NIL	N/A	N/A	NIL	Resolved
17	CML	RT‐PCR	70‐79	M	Y	Dasatinib	61	Imatinib	NIL	Resolved
18	ET	RT‐PCR	70‐79	M	Y	Hydroxycarbamide and Anagrelide	1	N/A	O_2_	Resolved
19	Myelofibrosis	RT‐PCR	60‐69	M	Y	Ruxolitinib	1	N/A	Intubation	Death
20	PRV	CT	50‐59	F	Y	Hydroxycarbamide	1	N/A	O_2_	Resolved
21	Myelofibrosis	RT‐PCR	70‐79	F	NIL	NIL	N/A	N/A	O_2_	Death
22	CNS lymphoma	RT‐PCR	70‐79	F	Y	MATRix & Auto HSCT	N/A	N/A	O_2_	Ongoing
23	DLBCL	RT‐PCR	70‐79	F	Y	R‐CHOP	5	N/A	NIL	Death
24	DLBCL (RS)	RT‐PCR	70‐79	M	Y	R‐CHOP	9	N/A	CPAP	Death
25	DLBCL PTLD	CT	60‐69	F	Y	Rituximab	10	N/A	Intubation	Death
26	FL	RT‐PCR	70‐79	M	Y	Rituximab maintenance	56	R‐CHOP	NIL	Death
27	MCL	RT‐PCR	70‐79	M	Y	Ibrutinib and Venetoclax	4	R‐CHOP	O_2_	Death
28	PTCL NOS	RT‐PCR	≤49	M	Y	CHOP	39	N/A	O_2_	Resolved
29	PTCL NOS	RT‐PCR	60‐69	M	Y	CHOP	15	N/A	O_2_	Death
30	WM	RT‐PCR	50‐59	F	Y	Ibrutinib	1	R‐CP	O_2_	Resolved
31	WM	RT‐PCR	50‐59	M	Y	Rituximab maintenance	UNK	UNK	NIL	Resolved
32	DLBCL	RT‐PCR	50‐59	M	NIL	NIL	N/A	R‐CHOP	CPAP	Ongoing
33	DLBCL	RT‐PCR	50‐59	F	NIL	NIL	N/A	R‐CHOP, R‐ESHAP	NIL	Resolved
34	DLBCL	RT‐PCR	≥80	F	NIL	NIL	N/A	N/A	NIL	Resolved
35	FL	RT‐PCR	60‐69	F	NIL	NIL	N/A	Radiotherapy	O_2_	Resolved
36	MZL	RT‐PCR	70‐79	M	NIL	NIL	N/A	N/A	CPAP	Resolved
37	CLL	RT‐PCR	70‐79	M	Y	Venetoclax	UNK	UNK	O_2_	Death
38	CLL	RT‐PCR	≥80	F	Y	Ibrutinib	1	Ofatumumab and Bendamustine	NIL	Resolved
39	CLL	RT‐PCR	70‐79	M	NIL	NIL	N/A	N/A	CPAP	Death
40	CLL	RT‐PCR	≥80	M	NIL	NIL	N/A	N/A	NIL	Death
41	CLL	RT‐PCR	60‐69	M	NIL	NIL	N/A	N/A	O_2_	Resolved
42	CLL	RT‐PCR	70‐79	M	NIL	NIL	N/A	N/A	O_2_	Resolved
43	CLL	RT‐PCR	60‐69	M	NIL	NIL	N/A	N/A	O_2_	Resolved
44	CLL	RT‐PCR	70‐79	F	NIL	NIL	N/A	N/A	O_2_	Resolved
45	CLL	RT‐PCR	70‐79	F	NIL	NIL	N/A	N/A	CPAP	Resolved
46	CLL	RT‐PCR	≥80	M	NIL	NIL	N/A	Ofatumumab and Chlorambucil	Nil	Resolved
47	CLL	RT‐PCR	70‐79	M	NIL	NIL	N/A	N/A	O_2_	Resolved
48	Myeloma	RT‐PCR	70‐79	F	Y	DVD	3	VTD	O_2_	Death
49	Myeloma	RT‐PCR	70‐79	M	Y	Carfilzomib	14	N/A	O_2_	Resolved
50	Myeloma	RT‐PCR	50‐59	M	Y	KRD	UNK	VTD, ASCT	CPAP	Death
51	Myeloma	RT‐PCR	≥80	M	Y	CTDa	65	N/A	O_2_	Death
52	Myeloma	RT‐PCR	≥80	M	Y	Daratumumab	4	CTD, Lenalidomide, Bortezomib	Intubation	Death
53	Myeloma	RT‐PCR	≥80	F	Y	DVD	6	Bortezomib	NIL	Death
54	Myeloma	CT	≤49	F	Y	Isatuximab, Pomalidomide, Dexamethasone	5	VTD, DT‐PACE, Autologous HSCT, Allogeneic HSCT, IRD	O_2_	Death
55	Myeloma	RT‐PCR	50‐59	M	Y	Panobinostat, Bortezomib, Dexamethasone	6	Carfilzomib, IRD, Pomalidomide, CC92480, Belantamab mafodontin	NIL	Resolved
56	Myeloma	RT‐PCR	≥80	F	Y	Pomalidomide, Dexamethasone	7	LCD, VCD, CTDa	O_2_	Death
57	Myeloma	RT‐PCR	≥80	M	Y	VCD	14	N/A	NIL	Death
58	Myeloma	RT‐PCR	70‐79	F	Y	VTD	3	N/A	O_2_	Death
59	Myeloma	RT‐PCR	≥80	M	Y	Lenalidomide	5	N/A	O_2_	Death
60	Myeloma	RT‐PCR	50‐59	M	NIL	NIL	N/A	Bortezomib, DVD	O_2_	Death
61	Myeloma	RT‐PCR	≥80	M	NIL	NIL	N/A	N/A	O_2_	Ongoing
62	Myeloma	RT‐PCR	≥80	F	NIL	NIL	N/A	VCD	CPAP	Resolved
63	Myeloma	RT‐PCR	≥80	M	NIL	NIL	N/A	N/A	O_2_	Ongoing
64	Myeloma	RT‐PCR	≥80	M	NIL	NIL	N/A	N/A	O_2_	Resolved
65	T‐LGL	RT‐PCR	60‐69	M	NIL	NIL	N/A	N/A	O_2_	Resolved
66	T‐LGL	RT‐PCR	60‐69	F	NIL	NIL	N/A	N/A	NIL	Resolved

Abbreviations: AML, acute myeloid leukaemia; CHOP, cyclophosphamide, doxorubicin, vincristine and prednisolone; CLL, chronic lymphocytic leukaemia; CML, chronic myelomonocytic leukaemia; CT, computed tomography; CTDa, attenuated cyclophosphamide, thalidomide and dexamethasone; DA, daunorubicin and cytarabine; DLBCL, diffuse large B‐cell lymphoma; DT‐PACE, dexamethasone, thalidomide, cisplatin, doxorubicin, cyclophosphamide and etoposide; DVD, daratumumab, bortezomib and dexamethasone; ET, essential thrombocythaemia; FCR, fludarabine, cyclophosphamide and rituximab; FL, follicular lymphoma; HSCT, haematopoietic stem cell transplant; IRD, ixazomib, lenalidomide and dexamethasone; KRD, carfilzomib, lenalidomide and dexamethasone; LCD, lenalidomide, cyclophosphamide and dexamethasone; MATRix, methotrexate, cytarabine, thiotepa and rituximab; MCL, mantle cell lymphoma; MDS, myelodysplastic syndrome; MPN, myeloproliferative neoplasm; MZL, marginal zone; N/A, not applicable; PRV, polycythaemia rubra vera; PTCL NOS, peripheral T‐cell lymphoma; PTLD, post‐transplant lymphoproliferative disorder; R, rituximab; R‐CP, rituximab, cyclophosphamide and prednisolone; RS, Richter Syndrome; RT‐PCR, reverse transcriptase polymerase chain reaction; SARS‐CoV‐2, severe acute respiratory syndrome coronavirus 2; T‐LGL, T‐cell large granular lymphocytic leukaemia; T‐MDS, therapy‐related MDS; UNK, unknown; VTD, bortezomib, thalidomide and dexamethasone; WM, Waldenström's macroglobulinaemia.

The pCFR was significantly higher amongst patients receiving immunosuppressive or cytotoxic treatment in the last 3 months compared to those who had not: 26 of 37 (70%) versus 8 of 29 (28%) *P* = .0013, with difference in pCFR 0.426 ± 0.184 (90% confidence interval, 2 proportions *Z* test) (Figure 2). It is not possible to determine from our data if treatment itself increases the risk of mortality, or if other confounding factors might explain this effect. The median ages of the two populations, one important potential confounder, were very similar at 74 years for the group with recent treatment and 73 years for the group not receiving treatment (not statistically significant).

Examining outcomes according to modality of treatment, only 6 patients had received anti‐CD20 therapy in the past 2 years, of whom 4 died. No patients had received purine analogues in the past 2 years. One patient in the cohort was 27 months post‐allogeneic stem cell transplant, having relapsed and received further myeloma therapy. This patient did not survive. A further 2 patients were within 2 years of autologous stem cell transplant, one with myeloma who did not survive and one with primary central nervous system lymphoma, who recovered.

## 4 DISCUSSION

At present, the true incidence of COVID‐19 in patients with haematological malignancy is not known, since only those with symptoms sufficient for hospital admission, or those already admitted who developed symptoms, were tested, in line with national guidance at the time. The true case fatality rate of COVID‐19 in this patient group is therefore likely to be significantly lower than that reported in this paper. In line with other studies, advancing age appears to be a key correlate of poor outcome in patients hospitalised with COVID‐19 infection, with a pCFR amongst those with haematological cancers of 59.5% in those over 70 years, as compared to 37.5% in those under 70 (Figure 1).

A relevant question is what proportion of the regional population with haematological malignancy these 66 patients represent. Hospital data on the numbers of patients fulfilling the UK government's criteria for shielding were available from 2 hospitals covering a total of population of 1.27 million. In these hospitals, 3334 patients were identified, equivalent to 263 per 100 000 population, which accords with the UK Haematological Malignancy Research Network prevalence data indicating a prevalence of 167/100 000 for haematological malignancy diagnosed in the last 3 years or 388/100 000 diagnosed in the last 10 years.^13^ We therefore estimate a regional population of between 4670 and 10 850 patients with haematological malignancy, indicating the only a small proportion (0.6%‐1.4%) of patients were admitted with SARS‐CoV‐2 infection in the period of this series.

It is striking to see that patients receiving chemotherapy within the 3 months preceding their COVID‐19 diagnosis have a statistically significantly higher pCFR (62%) than those who have not recently had chemotherapy (28%) (Figure 2). This may reflect the immunosuppressive effects of the chemotherapy, or of the underlying condition itself, or may reflect the increased frailty associated with active malignancy. The relatively small number of cases involved precluded formal multivariate analysis of potential confounding factors, and it is therefore not possible to determine whether receiving therapy is an independent risk factor for mortality. Equally the small numbers of patients receiving any one treatment or class of treatments precludes analysis of the effect of specific treatment types on outcome.

A further important point is that a proportion of this patient group will have a limited prognosis from their haematological malignancy or comorbidities. Of the 37 patients who had received cytotoxic or immunosuppressive treatment, only 8 could be classified as being given with curative intent. Of the other 29 patients, 9 would be expected to give relatively durable disease control, for example first‐line treatment of follicular lymphoma or myeloma; tyrosine kinase inhibitors for chromic myeloid leukaemia; or cytoreduction for polycythaemia vera or essential thrombocythaemia. In the remaining 20 patients, prognosis would be expected to be more limited and 16 of the total 34 deaths occurred in this group. Such considerations would also have been relevant to discussions between clinicians and patients regarding the appropriateness of intensive care unit admission or intubation and ventilation. Only 3 of the 34 patients who died were intubated prior to death and in the other 31 cases, a decision not to undertake intubation and ventilation was made in discussion with the patient, or their family where necessary, frequently with involvement of a clinician with intensive care expertise.

In our region, 50‐60 allogeneic stem cell transplants are performed each year, and the service is centralised to one centre. It is striking that only a single patient post‐allogeneic stem cell transplant was admitted with SARS‐CoV‐2 infection. This patient was 2 years post‐transplant and had relapsed and received subsequent myeloma therapy. We are aware of only 1 other patient in our region diagnosed with SARS‐CoV‐2 in the post‐allogeneic transplant setting who had mild symptoms only and was not admitted to hospital. This low rate of infection may be related to patterns of behaviour and strict adherence to shielding measures in this population, as this population is clearly highly immunosuppressed.

These data do indicate, however, that patients with haematological malignancies requiring hospital admission have a high mortality rate, supporting measures to minimise the risk of SARS‐CoV 2 exposure in this patient group. The finding is also in agreement with preliminary data from a very large population cohort study of 17 425 445 adult patients in England; the pre‐print data from which suggest a diagnosis of haematological malignancy within 5 years have been associated with at least a 3 times greater risk of death in hospital from COVID‐19 during the period 01 February to 25 April 2020.^14^ A subsequent UK observational study of 800 patients with cancer included 167 patients with haematological malignancy.^15^ Although the risk of death was not significantly increased in patients with haematological malignancy as compared to other cancers in this population with diagnosed infection, it is striking how high a proportion of included patients had a haematological diagnosis, although it is unclear whether this relates to increased risk of infection, risk of developing more severe disease or likelihood of testing. Interestingly, this study did not find a difference in mortality between those who had received cancer treatment in the last 4 weeks and those who had not, although this analysis was of the entire population including non‐haematological cancers and the treatments given were very heterogenous.

Our data support attempts to reduce the contact of individuals from this group with the healthcare system to minimise nosocomial infections.^16^ This group of patients need to be prioritised in consideration of how best to use a SARS‐CoV‐2 vaccine, and included in clinical trials of novel therapies to treat COVID‐19.

The submission of patient data to national and international databases is strongly encouraged, since numbers are insufficient at present to answer the questions that clinicians and patients alike are posing, regarding the relative risks of different diagnoses and treatments. These more complete data will, in time, be fundamental in enabling us to build an informed consensus about future management of haematological malignancy in the era of COVID‐19.
